# LIVING WITH MULTIMORBIDITY: SHARED EXPERIENCES OF PATIENTS, FAMILY CAREGIVERS, AND HEALTHCARE PROFESSIONALS

**DOI:** 10.1093/geroni/igac059.1854

**Published:** 2022-12-20

**Authors:** Anna Peeler, Katie Nelson, Sarah Badawi, Lara Street, David Hager, Patricia Davidson, Binu Koirala

**Affiliations:** Johns Hopkins University School of Nursing, Baltimore, Maryland, United States; Johns Hopkins University, Baltimore, Maryland, United States; Johns Hopkins University, Baltimore, Maryland, United States; Johns Hopkins Hospital, Baltimore, Maryland, United States; Johns Hopkins Hospital, Baltimore, Maryland, United States; University of Wollongong, Wollongong, New South Wales, Australia; Johns Hopkins University, Baltimore, Maryland, United States

## Abstract

Over 50% of patients in intermediate care units (IMCU) present with multimorbidity, two or more chronic conditions. Balancing the effects of multimorbidity and their treatments with quality-of-life can be a challenge. This experience-based co-design project aimed to elicit experiences of patients, family caregivers, and healthcare professionals in IMCU, in the context of challenges and intricacies of multimorbidity management, to inform the development of a symptom management toolkit. Patients aged 55 years and older were recruited and interviewed in person. Healthcare professionals working in IMCU (i.e., physicians, nurses, respiratory therapists, social workers, etc.) were recruited and interviewed virtually. Participants were asked questions about their role in recognizing and treating symptoms, factors affecting quality of life, symptom burden and trajectory over time, and symptom management strategies that have and have not worked. An inductive thematic analysis approach was used for data analysis. Twenty-three interviews were conducted: 9 patients, 2 family-caregivers, and 12 healthcare professionals. Patients’ mean age was 67.5 (± 6.5) years, over half (n=5) were Black or Hispanic, and average number of multimorbidity was 3.67. Five major themes emerged: 1) importance of patient-provider relationship; 2) open and honest communication; 3) accessibility of resources during hospitalization and at discharge; 4) caregiver support, training, and education; and 5) care-coordination and follow-up care. Patients, caregivers, and healthcare professionals often have different priorities for multimorbidity management, treatment, and education. However, given the growing population of patients experiencing multimorbidity, it is imperative to identify shared priorities and target holistic interventions considering their experiences to enhance outcomes.

